# Advanced Nanotechnology for Enhancing Immune Checkpoint Blockade Therapy

**DOI:** 10.3390/nano11030661

**Published:** 2021-03-08

**Authors:** Chiara Cremolini, Emanuela Vitale, Raffaella Rastaldo, Claudia Giachino

**Affiliations:** 1 Department of Translational Research and New Technologies in Medicine and Surgery, University of Pisa, 56126 Pisa, Italy; chiara.cremolini@unipi.it; 2 Department of Clinical and Biological Sciences, University of Torino, 10043 Orbassano, Italy; emanuela.vitale@unito.it (E.V.); claudia.giachino@unito.it (C.G.)

**Keywords:** cancer therapy, immunotherapy, nanomedicine, nanoparticles, active targeting, nanoconjugates, immune checkpoint receptor

## Abstract

Immune checkpoint receptor signaling pathways constitute a prominent class of “immune synapse,” a cell-to-cell connection that represses T-lymphocyte effector functions. As a possible evolutionary countermeasure against autoimmunity, this strategy is aimed at lowering potential injury to uninfected cells in infected tissues and at minimizing systemic inflammation. Nevertheless, tumors can make use of these strategies to escape immune recognition, and consequently, such mechanisms represent chances for immunotherapy intervention. Recent years have witnessed the advance of pharmaceutical nanotechnology, or nanomedicine, as a possible strategy to ameliorate immunotherapy technical weaknesses thanks to its intrinsic biophysical properties and multifunctional modifying capability. To improve the long-lasting response rate of checkpoint blockade therapy, nanotechnology has been employed at first for the delivery of single checkpoint inhibitors. Further, while therapy via single immune checkpoint blockade determines resistance and a restricted period of response, strong interest has been raised to efficiently deliver immunomodulators targeting different inhibitory pathways or both inhibitory and costimulatory pathways. In this review, the partially explored promise in implementation of nanotechnology to improve the success of immune checkpoint therapy and solve the limitations of single immune checkpoint inhibitors is debated. We first present the fundamental elements of the immune checkpoint pathways and then outline recent promising results of immune checkpoint blockade therapy in combination with nanotechnology delivery systems.

## 1. Introduction

Immunotherapy is a modern branch of oncotherapy modulating immune system activity against cancer. Starting from recombinant cytokine treatment, immunotherapy consists of several therapeutic approaches such as cancer vaccines, monoclonal antibodies (mAb) as well as cellular and gene therapies [1,2,3,4,5].

In recent years, cancer immunotherapy progression has been strongly driven by the study of immune checkpoint proteins (ICPs) as targets to restore and rejuvenate the immune response and T-cell activity in cancer. The majority of ICPs under physiological conditions limit and control T-cell activity, driving homeostasis processes and preserving self-tolerance; however, during cancer progression they are modulated in altered ways, favoring tumor escape and tumor growth. Both innate and adaptive immune cells can express ICPs that directly or indirectly orchestrate the crosstalk with cancer cells, thus producing a network in which mechanisms such as the dysfunction and exhaustion of T cells and the metabolic alteration of immune cells converge into immune suppression. The most studied ICPs are cytotoxic-T-lymphocyte-associated protein 4 (CTLA-4), program death 1 (PD-1) and PD-ligand 1 (PD-L1). Starting in 2011, the Food and Drug Administration (FDA) has approved mAbs blocking ICPs (immune checkpoint inhibitors (ICIs)), which have a strong clinical impact in solid tumors and deeply improved the prognosis of some of them. This is the case for tumors bearing microsatellite instability independently of their site of origin, whose sensitivity to ICIs led to the first tissue-agnostic drug approval by the FDA [6]. Meaningful results were obtained in solid tumors, including non-small-cell lung cancer, renal cell carcinoma, melanoma, head-and-neck tumors, urothelial cancer and hematological diseases such as Hodgkin lymphoma [7]. More controversial findings were achieved in other malignancies, where the therapeutic efficacy of ICIs seemed restricted to subgroups of patients, such as in the case of esophagogastric tumors or breast cancer [8,9]. Finally, disappointing results were reported with the use of ICIs in cancers with immune-excluded or immune-desert microenvironments, including colorectal tumors where several combination strategies are under investigation in clinical trials [10].

Overall, also in the case of immune-competent tumors like non-small-cell lung cancer or melanoma, where ICI efficacy is clinically relevant, some patients are not sensitive at all to this approach, and others experience disease progression after an initial, more or less durable response. This is due to the high levels of inter- but also intra-tumor variability with regard to multiple mechanisms of primary and acquired resistances, such as inefficient antigen presentation, poor T-cell infiltration and tumor mutational burden [11,12,13].

Currently, pharmaceutical nanotechnology, or nanomedicine, is applied in onco-therapy to deliver cytotoxic drugs into the tumor mass [14]. Nanotechnology is an interdisciplinary science and represents an effective tool to design highly effective combinational therapies boosting the effectiveness of immunotherapy and overpowering its limits. Nanoparticles (NPs) are designed in multiple ways, thanks to their plasticity in composition, geometry and size and may be useful to overcome critical points of ICI therapy, such as the localized and controlled ICI release, availability and ICI stability after infusion; further, they may allow for a reduction of ICI dose and control over adverse immune-related events (AIEs). NPs preferentially reach the tumor site via an enhanced permeability and retention (EPR) effect, a passive process mainly based on higher permeability of the tumor-associated blood vessels and ability to accumulate the nano-sized delivery systems [15]. However, the EPR may not be the only pathway driving the NPs to enter solid tumors as trans-endothelial, active processes have been recently implicated [16] that might be relevant in the human setting. Elucidation of the molecular mechanisms possibly involved in these active mechanisms of NP extravasation will be important, as will be the understanding of the role of surface adsorbed proteins during NP–tumor endothelial cell interaction. Additionally, NPs can be engineered to aim at active cellular targeting, and NPs of small dimensions can cross physiological blockades, like the blood brain barrier (BBB) [17,18], and reach the complex tumor microenvironment (TME) [19], thus improving drug delivery. Generally, NPs consist of a core, in which drugs are concentrated, and a shell, the functionalizable external layer. This structure allows multiple drugs to be simultaneously carried and target delivered, thus supporting combinatorial therapeutic strategies. The combination of immunotherapy and nanomedicine may enhance the efficacy of single conventional therapy [14,20,21,22]. Here we summarize preclinical studies proposing this approach, focusing on CTLA-4 and PD-1/PD-L1 blockade therapy, both as single agents and in combination strategies.

## 2. Immune Checkpoint Proteins in Brief

Checkpoint molecules exert a prominent role in the initial T-cell activation event at the level of lymph nodes, in the second activation within tissues, and also in the phenomenon of T-cell exhaustion. An important element is that in spite of a few overlaps in inhibitory functions, each checkpoint protein also covers distinct roles.

### 2.1. CTLA-4

CTLA-4 is one of the most studied inhibitory receptors. In conventional T cells it is expressed in the first phase of activation and by binding CD80 and CD86 costimulatory molecules, antagonizes the CD28 activation pathway [23]. Its surface expression is controlled by rapid endocytosis, recycling and degradation mechanisms [24]. Conversely, its constitutive expression in regulatory T cells (Tregs) controls their immunosuppressive activity.

CTLA-4 is principally implicated in regulation of T-cell activation in both lymph nodes and tissues, as well as in Treg-mediated suppression of dendritic cell (DC) activity. However, it is not expressed on natural killer (NK) cells, and as a consequence it is not involved in the regulation of NK-cell functions. It is also associated with decreased circulating B cell numbers and antibody levels.

The lethal autoimmune phenotype observed in *CTLA-4* knockout mouse exemplifies its prominent function in priming and tolerance to self-antigens [25,26]. While mice bearing heterozygous CTLA-4 mutations do not show an obvious phenotype, in humans these mutations result in either impaired interaction of CTLA-4 with its ligands (CD80 and CD86) or CTLA-4 haploinsufficiency, which are associated with a predisposition for autoimmune disorders and immune dysregulation syndrome [27,28]. Moreover, a progressive loss of circulating B cells has been highlighted in patients with the CTLA-4 mutation, likely due to Treg dysfunction [27,28,29].

### 2.2. PD-L1/PD-1 Axis

PD-1 receptors are found expressed on activated T cells and NK cells, and consequently they regulate T-cell activation at the level of lymph nodes and tissues, NK-cell activity and cell differentiation into Tregs. The PD-1 inhibitory receptor is expressed on T cells upon antigen recognition via T cell receptor major histocompatibility complex (MHC) interaction; by binding PD-L1 and PD-L2 ligands, it directs a negative signal that breaks T-cell activation. In physiological conditions this mechanism limits and controls T-cell activity, driving homeostasis processes and preserving self-tolerance [30,31]. The chronic antigen exposure, as occurs in chronic infection and cancer, maintains a high level of PD-1 on T cells, and the engagement of PD-1/PD-L1 interactions promotes T-cell exhaustion and dysfunction. Following PD-L1 binding, PD-1 is phosphorylated on two intracellular tyrosine domains that recruit the tyrosine phosphatase SH2, counteracting T cell receptor and CD28 pathways through the dephosphorylation of zeta-chain-associated protein kinase 70 (Zap 70) and phosphatidylinositol-3-kinase (PI3K) [32]. This results in the exhaustion of T cells, which progressively loose proliferation, cytokine secretion and effector function abilities. An autoimmune phenotype is also described in *PD-1* knockout mouse, yet with a delayed onset and reduced severity compared with phenotype of *Ctla-4*^−/−^ mouse; these data suggest that the fundamental role of PD-1 is induction of tolerance to self-antigens and priming, but that it essentially regulates the immune responses in peripheral tissues [33,34,35].

The largely expressed ligand for PD-1, PD-L1, is expressed on T and B cells, DCs, macrophages, as well as several nonhematopoietic cell types, including epithelia, pancreatic islet cells, endothelial cells, fibroblastic reticular cells, astrocytes, neurons and also on cells at sites of immune privilege (like placental trophoblasts and pigment epithelial cells in the retina). The second ligand, PD-L2, has a much more restricted expression profile and is expressed essentially on DCs and activated macrophages [31,36,37,38,39].

### 2.3. TIGIT, TIM-3 and LAG-3

T cell immunoreceptor with immunoglobulin and immunoreceptor tyrosine-based inhibitory motif domains (TIGIT) receptors are found expressed on T cells upon activation and on NK cells. These receptors regulate T- and NK-cell activation at the level of tissues and modify the profile of DC-expressed cytokines from Th1 to Treg promoting. *Tigit*^−/−^ mouse shows a milder phenotype, indicating a minor implication of TIGIT in the induction of central tolerance to self-antigens while underlying its relevance in peripheral tolerance [40]. T cell immunoglobulin mucin-3 (TIM-3) receptors have a more widespread expression profile, including activated T cells, NK cells, monocytes and DCs. Their major role is stimulating the expansion of immune suppressor cells including Tregs, myeloid-derived suppressor cells (MDSCs) and macrophages and inhibiting the activity of effector T cells and NK cells in the peripheral tissues [41,42]. Finally, lymphocyte-activation gene 3 (LAG-3), also called CD223, is expressed in activated T cells, NK cells, plasmacytoid DCs and B cells. It promotes the expansion of Tregs and inhibits the activity of effector T cells and NK cells. A study on CD4+ cells demonstrated that LAG-3 modulates T-cell homeostasis. In particular, LAG-3 modulates sensitivity to Treg suppression by limiting signaling via signal transducer and activator of transcription 5 (STAT5) phosphorylation. In addition, LAG-3 triggers forkhead box P3 (FOXP3)-induced Treg differentiation [43]. The phenotypes of *Tim-3*^−/−^ and *Lag-3*^−/−^ mice are exceptionally mild, and either autoimmunity needs to be induced in the mice, or genetically permissible background is necessary, respectively, in order for the phenotype to be manifested [43,44]. Since LAG-3 KO did not show autoimmunity, LAG-3 could be a more appropriate target for immunotherapy.

## 3. Immune Checkpoint Blocker Delivery with Nanocarriers

**Figure 1:**
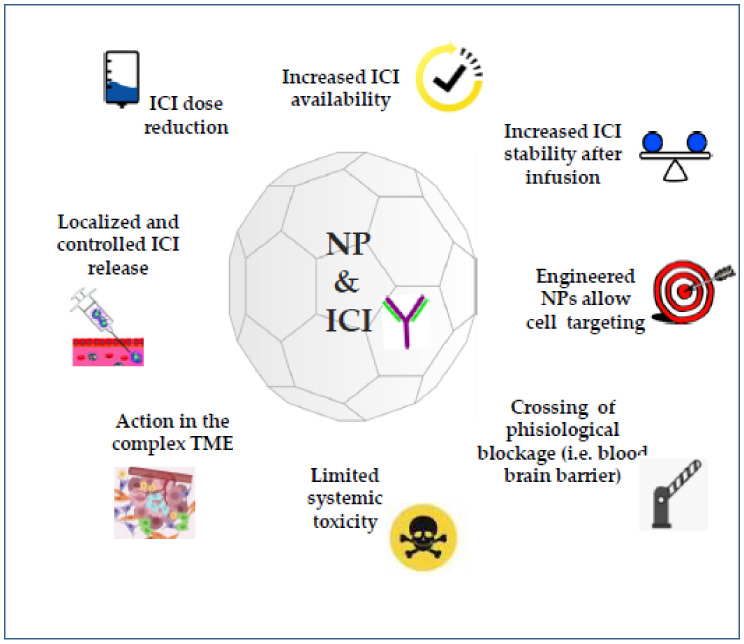
Combination of immune checkpoint inhibitors (ICIs) and nanomedicine. Nanoparticle (NP)-mediated ICI delivery enhances the efficiency and solves some limits of the single conventional therapy.

In the last few years, several NP systems have been optimized and applied to cancer immunotherapy (reviewed in [21]). Among a wide array, liposome, gold and polymer-based NPs are the most frequently used delivery systems for cancer immunotherapy [22,45]. NPs represent commonly used nanoparticle systems [45]. All these NPs can be promisingly used for delivering ICIs to the target site in an accurate and precise manner, thus overcoming some of the limitations of ICI therapy (Figure 1).

### 3.1. CTLA-4

The first antibodies that progressed into clinical trials after showing promising results in cancer treatment were ipilimumab, tremelimumab and quavonlimab, three anti-CTLA-4 antibodies. Ipilimumab is clinically employed for treatment of unresectable stage III/IV metastatic melanoma and as adjuvant therapy for radically resected "high-risk" melanoma patients [46,47,48,49], while tremelimumab and quavonlimab are currently being tested in clinical trials for safety and efficacy determination.

Although anti-CTLA4 treatment allowed to the overall survival of melanoma to be reached, with even cure of metastatic disease, for about 20% of the patients [46,47,50,51], nevertheless clinical application is limited because of its AIEs [52,53]. It is likely that CTLA-4 blockade can promote recruitment of peripheral T cells, thus enhancing the probability of autoimmune reaction [54]. A correlation between dose and both efficacy and toxicity has been highlighted. In addition, the efficacy of anti-CTLA-4 treatment as well as the AIEs are dependent on the time of exposure [55,56,57]. Clinical trials revealed up to 28% AIEs (Common Terminology Criteria for Adverse Events (CTCAE) grade 3–5) with systemic exposure to anti-CTLA-4 (3 mg/kg every 3 week) (that can increase up to 59% when combined to PD-1) [58]. These results confirm the need for nanotechnological delivery of ICIs, thus enhancing antibody bioavailability inside the tumor in order to improve the efficacy of the treatment, and at the same time limiting the toxic effects due to the systemic exposition.

NP-mediated ICI delivery seems an efficient and feasible approach to overcome the unsuccessful systemic administration of mAb against ICP in brain glioma treatment, due to drug failure in crossing the BBB. For this reason, Galstyan et al. combined nanotechnology and immunotherapy [17]. These authors covalently attached ICI mAb, such as anti-CTLA-4 (and/or anti-PD-1), to a biological polymeric scaffold made of poly(β-L-malic acid) (PMLA). These nanoscale immunoconjugates allowed the ICI mAb to go across the BBB and get to the cancer environment, where they modulated the immune response. In particular, glioblastoma (GBM)-bearing mice treated with nanoscale immunoconjugates exhibited augmented infiltrating T cells with anti-tumor activity and copious cytokine release. In addition, an increase in NK cells was accompanied by a decrease of Tregs at the tumor level. Moreover, mice bearing GBM showed an increase in survival when treated with nanocarriers loaded with anti-CTLA-4 (or anti-PD-1) with respect to the free mAb, and mice survival became even longer when treated with nanocarriers loaded with both ICI mAb [17]. Moreover, mice bearing GBM showed a significantly longer survival when treated with double checkpoint inhibitor-bearing nanocarriers compared to those treated with single checkpoint inhibitors. This study highlights that nanotechnology may be useful to deliver immunotherapy to primary brain tumor and brain metastasis, thus allowing the short lifespan of patients to be successfully extended.

As for possible nanotechnology-driven delivery, in vitro and in vivo studies were performed by Nikpoor and colleagues, who proposed two liposome formulations, one polyethylene glycol (PEG)ylated and the second non-PEGylated, containing anti-CTLA-4 blocking mAb in a C-26 colon cancer model [59]. Interestingly, the formulations accumulated at the tumor site within 18 h after injection; indeed, the best formulation was the PEGylated liposomes that, at this time point and after 24 h of treatment, were accumulated into the tumor seven-fold more than non-PEGylated and free mAb. Furthermore, the stability and greatest half-lives of PEGylate compound support the CTLA-4 prolonged circulation until 24 h. According to this distribution, PEGylated anti-CTLA-4 administration significantly delayed tumor growth when compared with other treatments. Moreover, the treatment best increased the median survival time and the percent increase of life span. The therapeutic effect was associated with increased percentages of CD8^+^ T cells at the tumor site, indicating that other subsets of T cells, including CD4+ T cells and Tregs, may have limited roles during CTLA-4 blocking antibody cancer therapy. Recently, similar results were obtained by Alimohammadi et al. in a murine B16 large established melanoma model where anti-CTLA-4 encapsulated in PEGylated liposomes improved its therapeutic effect compared to the non-liposomal anti-CTLA-4, because nanocarrier delivery allowed a marked Ab accumulation in tumor and a prolonged half-life in blood. The authors observed increased tumor size regression and improved mice survival when anti-CTLA-4 was encapsulated into liposomes with respect to free Ab. Moreover, an enhancement in the CD8^+^ cells and CD8^+^/Treg ratio in tumor-infiltrated lymphocytes was observed in mice receiving the anti-CTLA-4 encapsulated in nanocarriers [60]. These studies confirm the effectiveness of nanotechnology for ICI delivery, and therefore this approach can be applied to other tumor types. Since ICP expression (such as CTLA-4, PD-L1) increases following chemotherapy [61], the association of ICIs with chemotherapy is a promising strategy to overcome the immune system inhibitory effect of chemotherapy. Alimohammadi et al. demonstrated that anti-CTLA-4 encapsulated into PEGylated liposomes improved the anticancer efficacy when combined with doxorubicin encapsulated into liposomes (Doxil). In particular, anti-tumor efficacy was observed when Ab administration occurred before chemotherapy [60]. In fact, an enhancement in the CD8^+^/regulatory T cell ratio was observed only when liposomal anti-CTLA-4 was given before Doxil. Both enhancement in treatment efficiency and side effects limitation can be reached with the synergic approach combining ICI nanocarrier delivery with chemotherapy. These results confirmed that the antitumor efficacy is affected by both order and time of administration of each component in the combined therapy [62]. Moreover, chemotherapeutic compounds, such as oxaliplatin (OXL), can reverse the immune-suppressive state of TME into an immune-favorable one [62]. All these studies highlight that nanocarrier-mediated ICI delivery when combined with chemotherapy increase even more the antitumor efficacy of the therapy.

The investigation of the ICI-triggered pathway is useful because it can allow different antibodies to be combined. For instance, Chae and colleagues developed nanocarrier-mediated delivery of two ICIs (i.e., anti-CTLA-4 together with anti-PD-1) with a synergic effect; thus, this treatment showed a slower tumor growth and longer mice survival compared to the monotherapy [63].

### 3.2. PD-L1/PD-1 Axes

Probably the most favorable target for cancer immunotherapy has been the PD-1:PD-L1 pathway. Two anti-PD-1 antibodies (pembrolizumab and nivolumab), and three anti-PD-L1 antibodies (atezolizumab, avelumab and durvalumab) are currently approved for the therapy of several solid tumors [64,65,66,67,68,69,70,71,72,73,74,75,76,77,78,79,80,81,82,83]. Further, several other mAbs against PD-1 and PD-L1 are under clinical development. Along with very promising clinical results, evidence of AIEs arose also in this case, with inflammatory side effects showing heterogeneity in timing and organ involvement. For example, the general AIEs of anti-PD-1 in non-small-cell lung cancer patients occur within a few weeks to 3 months after treatment initiation, and nearly all significant organs can be affected. In some cases, the onset of AIEs, like pulmonary and hepatic toxicity, is delayed up to 1 year after treatment initiation [84].

Interestingly, nanotechnology can deliver combinations of ICIs, thus simultaneously targeting innate and adaptive immunity. Chen et al. engineered an immune-complex with reactive oxygen species (ROS) responsive cross-linker (aPD-1@aCD47 complex) [85]. Originally, the authors proposed a strategy not exclusively aimed at the immune checkpoint inhibitor delivery. Indeed, ROS responsive cross-linker ensured the ICI controlled release and the remodeling of TME by ROS captured through the complex. Interestingly, they reported that, in murine B16F10 melanoma tumor model, ROS complex significantly reduced the expression of matrix metalloprotease2 (MMP2), and both percentage of M2 tumor associated macrophages and Treg into tumor mass, thus increasing and favoring CD8^+^T lymphocytes. Importantly this modulation supports the immunotherapy efficacy. Indeed, when ROS complex was integrated with anti-PD-1 and anti-CD47 mAbs, under the rationale that blocking CD47 will activate phagocytic cells to phagocytize cancer cells and promote antigen presentation [86], melanoma growth was reduced in mice receiving aPD-1@aCD47 complex when compared to free antibody treated and untreated mice. Additionally, in the B16F10 model, aPD-1@aCD47 complex inhibited the growth of primary and metastatic tumor.

Nanotechnology is also useful to reduce drug dose; Schmid and colleagues interestingly proposed poly (lactide-o-glycolic) acid (PLGA) and PEG NPs conjugated with anti-PD-1 mAb (PD-1 targeting NPs) and loaded with the transforming growth factor-β receptor 1 (TGF-β R1) inhibitor, SD-208, in order to target PD-1^+^ cells and to block the immunosuppressive activity of TGF-β in an MC38 colon cancer model [87]. They showed that PD-1 targeting NPs-SD-208 acted on CD8^+^T cells, reduced tumor growth and ameliorated animal survival. The therapeutic effect was obtained at low doses of ICI (20 μg of anti-PD-1 and 40 μg of SD-208), whereas anti-PD-1 mAb and SD-208 in free administration did not have any effects. Interestingly, the effectiveness at lower dose can allow for the limiting of side effects. Importantly, the nanotherapy was able to induce T-cell activation both at the tumor site and in the periphery. Indeed, the treatment increased the number of granzymeB^+^ and interferon ɣ (INFɣ)^+^ CD8^+^T cells in the tumor mass and the percentage of circulating activated CD4^+^, CD8^+^ T cells, and NK cells. Schmid and colleagues demonstrated that PD-1 targeting NPs were also effective in a second combinatorial anti-cancer treatment aimed to modulate TME, thus favoring the local inflammation. Indeed, they loaded PD-1 targeting NPs with the agonist of toll-like receptors 7 and 8, namely R848. The therapeutic effect was similar to the previous NP preparation. The approach used in this study allowed for the targeting of tumor reactive PD-1^+^ T cells both in tumor and the periphery. Importantly, the anti-PD-1 antibody fragment conjugated to NPs displayed a double activity, driving NPs to T cells and blocking the inhibitor receptor PD-1.

Studying NP made of PLGA in which anti-PD-1 was encapsulated, Ordikhani et al. confirmed the effectiveness of the NP delivery of ICIs. Nevertheless, they highlighted the importance of dose selection in anticancer therapy. As a matter of fact, higher mortality in the B16-F10 murine melanoma model was observed at high dose of anti-PD-1 delivered with NPs with respect to free antibody. The authors attributed this unexpected toxicity to the over-activation of T cells mediated by secondary lymphoid tissues. In fact, the combined therapy-induced toxicity was completely reverted in splenectomized mice. Only a low dose of anti-PD-1 delivered with NPs allowed the maintenance of anticancer efficacy, which was four- to five-fold higher than free Ab or vehicle [88]. In the spleen, the anti-PD-1 NPs were uptaken by DCs, inducing their maturation, with consequent DC-mediated T cell-activation; additionally, the anti-PD-1 could mediate NP–effector cell interactions, thus increasing their adhesive capacity with cancer cells via induction of the increased expression of adhesion molecules [88]. The increased anticancer effectiveness of nanomedicine relies at least partially on nanocarrier accumulation in the tumor and sustained ICI release; notably, in another study, gold NP-mediated administration of anti-PD-1 allowed the ICI dose to be reduced to 1/5 [89].

Recently, Wu et al. proposed an alternative and multidisciplinary approach to block PD-1 and PD-L1 molecules with a nanotechnological approach aimed at preserving T-cell activity, such as tumor infiltrating T lymphocytes in a cell therapy strategy [90]. They used lipid-coated calcium phosphate NPs delivering siRNA against PD-1 and PD-L1, thus protecting their stability that in other delivery strategies is often compromised. In particular, these NPs have a core of calcium phosphate coated with a first layer of 1,2-dioleoyl-sn-glycero-3-phosphate and a second layer of lipids. Tumor infiltrating T lymphocyte, isolated and expanded from breast cancer patient expressed PD-1, were treated with LCN-siRNA PD-1 NPs, and their cytotoxic capability was tested in vitro against the MCF-7 cell line previously treated with LCN-siRNA PD-L1 NPs. Single silencing of PD-1 and PD-L1 and, more effectively, their combination improved the cytotoxic effect; indeed, at the middle ratio of 30:1 (effector:target) the percentage of killing efficacy of tumor infiltrating T lymphocytes to MCF-7 cells was 21% in the baseline condition and became 39% in the double siRNA condition, indicating that knocking down both immune checkpoint molecules led to a significant (*p* < 0.05) improvement of tumor infiltrating T lymphocyte antitumor effects. In double silencing, the cytotoxicity was associated with the increased release of IFN-ɣ and tumor necrosis factor-α (TNF-α). Wu’s study, even though limited by the exclusive in vitro analysis, displays an alternative starting point for immune regulation in chimeric antigen receptor (CAR) or T-cell therapy.

Wang and colleagues developed a groundbreaking approach consisting of a microneedle patch in which hyaluronic acid was combined with pH-sensitive dextran NPs carrying both the glucose oxidase/catalase (GOx/Cat) and the anti-PD-1 mAb [91]. The conversion of blood glucose into gluconic acid by the enzymatic component allowed a localized and prolonged ICI release due to their dissociation dependent on acid TME. This innovative approach allowed a release of the immunotherapeutics in a physiologically controlled manner. In an established melanoma model, the system significantly inhibited tumor growth and prolonged survival of animals, while the free intratumoral administration of anti-PD-1 mAb induced transient therapeutic effects followed by tumor relapse. The antitumor effect was associated with increased numbers of CD8+ tumor infiltrating T lymphocytes. Moreover, this innovative approach can be delivered in combination with other therapies, such as immunomodulators, thus increasing the efficacy of the treatment. In fact, the codelivery of anti-PD-1 and anti CTLA-4 achieved long term free survival in 70% of treated animals.

In order to augment antitumor efficacy, Liu et al. developed a new class of liposomes (LPDp) that were dual responsive to pH and MMP with PD-L1 inhibitor conjugate combined with low-dose chemotherapy doxorubicin (DOX) [92]. The synergistic action of the chemotherapeutic drug and ICI allowed the dual responsive liposomes to reach the optimal tumor suppression efficiency of ∼78.7% in an in vivo murine B16F10 melanoma model.

Along similar lines, Wang et al. used the PD-L1 molecule as a target to drive drug compounds directly into gastric tumor [93]. They employed anti-PD-L1 conjugated liposomes (immunoliposomes) carrying OXL and microRNA-130a (miR-130a) (PD–miOXNP), chemotherapy drug and miRNA, inhibiting the invasion and migration processes, respectively. Thus, immunoliposomes are a multipurpose strategy that connects chemo, miRNA and immunotherapies. The authors demonstrated that immunoliposomes increased miOXNP cellular uptake in gastric cancer both in vitro and in vivo, thus favoring anticancer effects. Indeed, PD–miOXNP induced tumor cell apoptosis and reduced tumor cell invasive capability in vitro, correlating with proliferation blockade and higher tumor growth inhibition in vivo when compared with the other drug formulations, i.e., free and single administration of OXL and miR-130a or their combination. Importantly, while the free OXL administration induced severe toxic effects, the immunoliposomes were safe. Of note, recently, studies have described intrinsic effects of PD-L1 engagement in tumor cells through the mammalian target of rapamycin (mTOR) pathway, thus involving PD-L1 in metabolism, autophagy, cell growth and metastatic capability of tumor cells [94]. The anticancer effects of immunoliposomes proposed by Wang et al. could also be ascribed to PD-L1 signaling block on tumor cells [95]. The immunoliposome strategy relies on PD-L1 expression on tumor cells; thus, it is also eligible for others cancer types expressing this ICP molecule, and it could be used in alternative formulations of cancer drugs.

Recently, Zhou and colleagues developed a PLGA–PEG micelle able to co-deliver all-trans retinoic acid (ATRA) and PD-L1 mAb for applications in oral squamous cell carcinoma and oral dysplasia [96]. In vivo antitumor assays clearly suggested a superior therapeutic efficacy of the ATRA–PLGA–PEG–PD-L1 compared to free ATRA and demonstrated that CD8+ T cells were activated in TME after treatment.

The challenging task in preparing nanocarriers capable of reacting to the multiple stimuli of TME in order to deliver anti-PD-1/PD-L1 antibodies together with chemotherapeutic agents in a spatio-temporally controlled manner was recently addressed by Su et al. [97]. They described a polymeric micelle with dual-sensitivity that can co-release anti-PD-1 Ab and the chemotherapeutic drug paclitaxel (PTX) in a controlled way, so that the two drugs can act synergistically on their own targets for an improved therapy. As micelles were decorated with anti-PD-1 antibodies via MMP-2 sensitive peptide linker, they could spatio-temporally direct the delivery of anti-PD-1 and PTX by reacting to the MMP-2 molecules being enriched in tumor tissue and lysosomal acidity of tumor cells, respectively. Both in vitro and in vivo experiments in a murine melanoma model were performed and demonstrated a potent antitumor effect likely due to a synergistic effect of immunotherapy and chemotherapy.

In another approach employing nanocarriers that could spatio-temporally deliver anti-PD-1 checkpoint inhibitors and agonistic anti-OX40 antibodies simultaneously to mouse 4T1 breast cancer, significant elevation of T-cell stimulation via enhanced release of IFN-γ and increased CD8+:Treg cell ratio was obtained, leading to doubled survival rates [98].

### 3.3. TIGIT, TIM-3 and LAG-3

The clinical development of anti-TIGIT, -TIM-3 and -LAG3 antibodies is currently being pursued by several pharma companies. Recently a few groups started to pre-clinically investigate new strategies of nanomedicine, applying these Abs in cancer immunotherapy. Since human acute myeloid leukemia (AML) cells express high levels of TIM-3 receptor [99], Yasinska et al. designed a nanocomplex formed by AuNPs carrying on their surface Abs against TIM-3 in order to target AML cells [100]. It is already known that rapamycin inhibits mTOR kinase activity leading to AML cell death [101]. However, when the surface of each AuNP was covered with rapamycin esterified with glutathione (GSH) ester, the treatment of leukemia cells failed [100]. On the contrary, a synergic effect on mTOR activity was observed when the nanocarrier was coated with both molecules, an effect that can be explained by the fact that rapamycin can induce the inhibition of mTOR activity inside the cells only after GSH cleavage by enzymes associated with the cell membrane. Moreover, the mechanism of delivery of rapamycin by this nanocarrier allowed an otherwise toxic drug dose to be supplied into AML cells, with a high level of efficiency targeting the TIM-3 receptors. In fact, a similar effect was obtained using a 50 times higher concentration of free rapamycin. Further studies are required to evaluate the effectiveness of this nanocomplex in an in vivo model of leukemia.

Recently, it has been reported that the TIM-3 pathway is involved in disabling of anti-cancer immune surveillance, not only in human myeloid leukemia [102], but also in several human cancer cell lines of both solid and liquid tumors (such as brain, colorectal, kidney, blood/mast cell, liver, breast, prostate, lung and skin tumors) [103]. Nanomedicine approaches based on Abs against TIM-3 receptor could thus be used to target different types of cancer cells.

However, the activation of the mTOR pathway was lower in breast cancer cells than in THP-1 human myeloid leukemia cells, thus suggesting the involvement of different pathways [103]. The delivery platforms previously described might be useful to treat different malignant tumors; further investigations are recommended for unveiling the pathway involved and identifying the most effective inhibitors to combine with TIM-3 Ab on the nanocarrier surface. It is likely that once an inhibitor of the pathway is identified, new immunotherapeutic anti-cancer strategies based on the suppression of the TIM-3 pathway can be designed, aiming at the eradication of different solid and liquid tumors by the immune system.

Despite no nanotechnology-driven delivery having been reported so far for anti-TIGIT and anti-LAG3, it is likely that further study might reveal their usefulness in nanomedicine. Indeed, the emerging clinical trial data pointed out their efficacy in the field of tumor immunotherapy. Promising antitumor activity of anti-LAG-3 in mice transplanted with colorectal cancer cells has been demonstrated, as well as the enhanced effectiveness when this treatment is combined with anti-PD-1 [104]. It is likely that the use of nanocarrier delivery systems can further improve their effects. Moreover, based on the relative absence of autoimmunity in LAG-3 KO mice, Durman et al. suggested a possible clinical advantage of LAG-3 blockade versus, for instance, CTLA-4 blockade in terms of decreased toxicity, thus rendering LAG-3 a suitable partner to block in cancer combination immunotherapy [43]. In light of the low autoimmunity levels observed in TIGIT KO mice, the same might turn out to be true also for this last ICP.

## 4. Combination of ICI with Nanocarrier-Mediated Therapies

**Figure 2:**
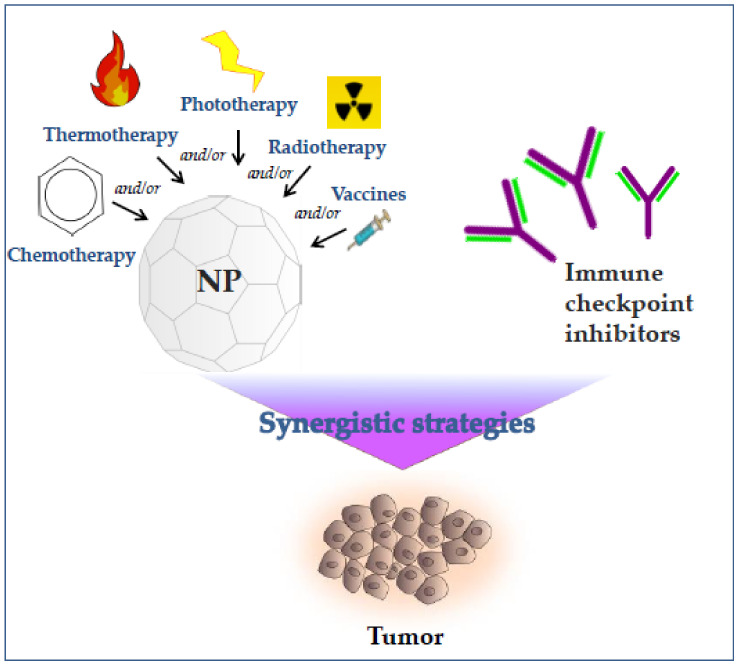
Synergistic nanomedical strategies improve the anticancer therapeutic effects of ICI. Nanoparticles (NP)-mediated delivery of chemo/photo/radio/thermo-therapy improves the antitumor effects via modulation of tumor microenvironment, enhancement of the innate immunity and relative antigen presentation, causing reduction of immunosuppressive effects. Their co-administration with ICIs shows a synergistic enhancement of the therapeutic effects.

Combination therapies represent useful synergistic strategies in which nanomedicine can intervene, activating the innate immunity and the relative antigen presentation, modulating the tumor microenvironment and reducing immunosuppressive effects. Additionally, nano-chemo/photo/radio/thermo products improve the delivery of anticancer drugs, and their association with ICIs enhances the therapeutic effects (Figure 2).

### 4.1. CTLA-4

Antigen availability and presentation are critical to activate the cancer immune response in order to induce tumor ablation with consequent tumor antigen release and to favor the innate immunity activation. Chen et al. investigated the therapeutic adjuvant efficacy of PLGA NPs encapsulating near-infrared (NIR) dye indocyanine green (ICG) and R837 in anti-CTLA-4 therapy for both breast and colon cancer [105]. They demonstrated the immune-stimulator activity of NPs; indeed, PLGA-ICG-R837 NPs induced maturation of DCs as both costimulatory molecule expression and capability to release IL-12, IL-6 and TNF-α in vitro and in vivo. Importantly, in photothermal therapy (PTT), the effects were significantly increased in the tumor mass and at the lymph node level; additionally, proinflammatory cytokines were detected at higher serum levels and for longer times than PLGA-ICG-R837 NP alone. Then, the authors went on to study the combination therapy of PTT-PLGA-ICG-R837 NPs and anti-CTLA-4 mAb in an artificial metastasis model of breast or colon cancer. Mice were subcutaneously inoculated with primary tumor and received the secondary tumor either subcutaneously or intravenously. Primary tumor was ablated by surgery or PTT. Only tumor ablation through PTT and associated with PLGA-ICG-R837 NPs assured the efficacy of ICI in metastasis treatment. Indeed, in the subcutaneous model, all mice were cured and did not show metastasis; additionally, in the intravenously model, 70% of the mice were cured, whereas the mice of all other groups died within 25–40 days due to metastasis overgrowth. The therapeutic effect was associated with increased CD8^+^ T and reduced Treg infiltration in the secondary tumors when compared with anti-CTLA4 mAb or PLGA-ICG-R837 single treatment. This was true also in an orthotopic breast cancer model. Interestingly the drug combination strategy proposed by Chen and colleagues was effective in inducing immunological memory. Indeed, rechallenged tumors inoculated 40 days post ablation of their first tumors, treated with PTT-PLGA-ICG-R837 NPs, had significant reduced growth when compared with all other combination strategies, and this was due to a persistently high percentage of effector memory CD8+ T cells. This study clearly underlined the need to support anti-CTLA-4 activity to obtain efficient therapeutic effects. Moreover, immune-adjuvant-carrying nanovectors offer vaccine-like functions in situ that foster a powerful antitumor immunity and in combination with ICI show efficacy in cancer immunotherapy. The ablation with PTT and the nanoparticle approach provided the essential vaccination effect to elicit T-cell activation and overcome the ICI limits. Of note, this therapeutic strategy employed low doses of anti-CTLA-4 mAb, thus reducing system toxic effects.

Not only in PTT but also in photodynamic therapy (PDT) and a radiotherapy setting does nanomedicine improve tumor antigen release and presentation, thus enhancing ICI efficacy. Nanocarrier-mediated PDT also augmented the efficacy of these therapies via reinforcement of cell destruction and consequent generation of tumor associated antigens in situ, thus allowing the inhibition of primary and metastatic tumor development by antitumor immune response [106]. These authors used upconversion NPs (UCNPs) for multi-task delivery. UCNPs contain lanthanide ions which allow NIR to be converted into visible radiation with higher energy and penetration depth into tissues [107]. UCNPs were simultaneously loaded with R837 and the photosensitizer, chlorin e6, in mice bearing CT26 colorectal cancer. Under NIR irradiation, a strengthened tissue penetration depth occurred, thus permitting PDT tumor destruction and tumor-associated antigen generation, which in the presence of the adjuvants (R837) carried by UCNPs, promoted a stronger antitumor immune response. Even in this case, a combination with CTLA-4 checkpoint blockade reinforced the antitumor efficacy and showed a long-term immune memory response to protect treated animals from rechallenged tumors. In another study, Chen et al. showed in an artificial model of metastatic colon cancer that the combination of radiotherapy and anti-CTLA-4 Ab partially delayed the growth of primary and distal subcutaneous tumor but did not show tumor inhibition [108]. They further combined radiotherapy and ICI with PLGA NPs encapsulating R837 and catalase (PLGA-R837@Cat). PLGA-R837@Cat, as a single agent, had strong immunostimulatory effects and induced the death of CT-26 tumor with consequent antigen release favoring the immunogenic cell death process (ICD) and DC maturation. Interestingly, catalase activity, by breaking down hydrogen peroxide (H_2_O_2_) into water and O_2_, significantly decreased hypoxia in the tumor mass; thus, TME was dramatically modulated. Indeed, the oxygenation of the microenvironment in turn reduced one of the most immunosuppressive sub-populations, the pro-tumoral M2 macrophages, and increased the percentage of proinflammatory M1 macrophages. When mice bearing primary and distal CT26 tumor were treated with radiotherapy, anti-CTLA-4 Ab and PLGA-R837@Cat, primary tumors were eliminated, and secondary tumors disappeared. The triple treatment allowed for impressive results that the authors did not obtain with all other combinations. Mechanistically, at an early time point, secondary tumor from mice treated with anti-CTLA-4 Ab and PLGA-R837@Cat had a higher number of CD8^+^ T cells when compared with those treated with single agents or other combinations. Furthermore, the authors observed a reduced percentage of CD4^+^ T cells corresponding to a decreased frequency of CD4^+^Tregs than what the radiotherapy setting generally induced. The approach proposed is a great example of synergic therapy in which the effects of each single agent support the other. The study shows that radiotherapy, PLGA-R837@Cat and anti-CTLA-4 Ab, allowed for the best results also in the breast cancer orthotopic model (4T1), with superior efficacy with respect to PLGA-R837@Cat+X-ray, surgery+anti-CTLA-4 and surgery+PLGA-R837@Cat. Metastases were strongly inhibited, and 60% of mice survived until 60 days from treatment start, while surprisingly no mice survived in the other groups.

Further, in a 4T1 tumor-bearing mice model, Chen et al. used iron-oxide nanoparticles (IONPs), which, after systemic administration, were strongly stable, and the small size favored their accumulation into the tumor site where they mediated hyperthermia after NIR irradiation. These authors suggested that local IONP-mediated photothermal therapy combined with ICI, such as anti-CTLA-4, can allow immunosuppression mediated by Treg to be overcome, thus boosting cancer immunotherapy [109].

### 4.2. PD-L1/PD-1 Axis

Due to their immunepotentiating capabilities, the combination of anthracyclines like epirubicin with PD-1/PD-L1 blockade is under intense clinical investigation [110]. Recent findings from Kinoh et al. demonstrate the ability of micelles loaded with epirubicin (Epi/m) to overcome anti-PD-1 resistance of phosphatase and tensin homologue (PTEN)-negative orthotopic GBM multiforme, typically containing large subpopulations of cancer stem cells and typically being highly resistant to ICIs [111]. These micelles, made of PEG-poly(aspartate-hydrazide) block copolymer, are capable of drug release in a pH-sensitive manner, and this feature leads to a reduction in immune-mediated cell death. The micelles were demonstrated to be capable of achieving antitumor effects in a synergistic manner with anti-PD-1 via the promotion of the ICD process; further, they decreased both the number of intramural MSDCs and PD-L1 expression on cancer cells, stimulating the future translation of the Epi/m plus anti-PD-1 combination into clinical trials. Nanomedicine is a promising tool for clinical practice because it can lead to converting “cold” tumors into “hot” ones. [111].

Recently, preclinical studies have focused on NPs delivering chemo or radiotherapy in combination with immunotherapy. Chemotherapeutics can stimulate antitumor T cell-responses. Kuai et al. showed that DOX-loaded nanodiscs caused tumor cell death without any side effects. In particular, the authors used pH-responsive nanocarriers, which promptly released DOX in response to pH 5, thus facilitating drug release in the acid pH of lysosomes/endosomes after nanodisc internalization into the tumor cells [112]. Nanodisc-mediated delivery of DOX potentiated priming of effector T cells directed towards a broadened tumor-associated epitope array, including whole tumor cells. The synergic effect of these combined therapies increased the antigen-specific cytotoxic T-cell response. Moreover, DOX-loaded nanodiscs potentiated PD-1 therapy, leading to a complete tumor regression in 80 to 88% of mice bearing MC38 and CT26 colon carcinoma and protecting survival animals against cancer relapse.

The cytotoxic activity of some recent onco-drugs induces ICD and proinflammatory processes, thus activating the immune response and breaking the immunosuppressive conditions and tolerance. ICD is primarily ascribed to the ROS. ICD modulates TME, thus supporting immunotherapy efficacy. Duan and colleagues [113] have recently proposed a nanocompound with a core of OXL and dihydroartemisinin (DHA) in the shell and studied it in a colon cancer model. This nanodrug induced cytochrome C release in a ROS-dependent way, with consequent apoptosis of colon cancer cells and additional blockade of tumor cell growth, arresting tumor cells in the G2/M phase. Interestingly, the resulting tumor cell apoptotic bodies were captured and presented by DC and macrophages. In vivo, only immunocompetent mice were protected by vaccination with colon cancer cells treated with OXL-DHA, underlining the immunogenicity effects of the treatment. OXL-DHA was assessed in vivo in combination with PD-L1 blockade in two colon cancer models, CT26 and MC38, the latter being highly immunosuppressive. Immunocompetent mice with established tumor mass were treated, and treatment led to eradication of 6/6 tumors in the CT26 model, with tumor-specific immune responses able to result in vaccination against subsequent live cell challenge. In the case of the highly immunosuppressive MC38 model, a higher dose of OXL-DHA needed to be employed in order to almost recapitulate the results observed in CT26, with tumor eradication in 3/5 mice and long-term tumor control in the other two.

Further, immunogenic “nano-scale coordination polymer” (NCP) particles constitute an innovative category of nanomaterials endowed with multimodality delivering properties, characterized by flexible composition and biodegradability within tissues. The core of this nanomaterial was obtained via phosphorylation of Zn^2+^ ions and OXL, then capped with a monolayer of 1,2-dioleoyl-*sn*-glycero-3-phosphate (DOPA) molecules, while the shell was mainly made of pyrolipid with a photosensitizer ability (NCP@pyrolipid), and it was employed to deliver chemotherapy and PDT to colorectal cancer in combination with anti-PD-L1 checkpoint inhibitors [114]. NCP@pyrolipid is a hybrid nanostructure that combines photosensitizers, oxygen and light to give rise to unstable ROS, in particular singlet oxygen (^1^O_2_) that are capable of efficiently destroying target tumor cells by promoting apoptosis and acute inflammation. As OXL-induced ICD had been previously demonstrated in colorectal cancer [115], a further three-level synergistic effect with (i) pyrolipid-induced PDT, (ii) OXL chemotherapy and (iii) immune checkpoint blockade was investigated. Treatment of murine colorectal cancers with a combination of anti-PD-L1 (pembrolizumab) and NCP@pyrolipid produced a 10-fold increase of CD8+ T-cell infiltrations in tumors [114]. In addition, incorporation of localized PDT produced an interesting effect on nonirradiated areas, as targeted tumors shrunk by 67%, while distant tumors regressed almost completely [114]. Other metastatic cancers may become targets of such a “triple combination”, provided that primary tumors are PDT-accessible. This possibility was in fact confirmed in two different (4T1 and TUBO) breast cancer murine models for a combination of anti-PD-L1 (pembrolizumab) with Zn-pyrophosphate (ZnP) NPs loaded with the photosensitizer pyrolipid (ZnP@pyrolipid) + PDT. The authors demonstrated an increased nanocomplex accumulation into the tumor, and they attributed this effect to nanocomplex that remained in systemic circulation for long periods of time. Beside the eradication of primary tumors, this therapeutic approach allowed the prevention of lung cancer metastasis and the suppression of non-irradiated pre-existing distant cancer boosting systemic antitumor immunity [116].

One of the major obstacles to immunomodulatory vaccines based on NPs is represented by the phagocyte tendency to sequester NPs, thus causing harmful accumulation in liver and spleen and poor target tissue attainment [117]. Luo et al. have described a flexible nanovaccine platform, conjugating a synthetic polymeric nanoparticle, PC7A, that acted as an immunogenic adjuvant leading to enhanced cross-presentation of antigens, antigen transport to lymph nodes and activation of “stimulator of interferon genes” (STING) pathways [118]. As a consequence, following uptake of PC7A, phagocytes become reprogrammed from “foe to friend.” In preclinical murine models, the nanovaccine proved effective in inhibiting melanoma and colon tumor proliferation [118]. In one of these tumor models, murine TC-1, the synergistic effect with PD-L1 blockade resulted in 100% survival with long-lasting effects after as many as 60 days, suggesting powerful anti-cancer memory [117,118].

An innovative blend of PD-L1 blockade, gold nanostars and laser light known as synergistic immuno photodermal nanotherapy (SYMPHONY) has been described to achieve some success [119]. The spiked geometry of gold nanostars directed their preferential accumulation inside tumor cells, where they functioned as “lightning rods” able to effectively capture and convert laser light energy into heat, in turn causing thermic death of tumor cells very deeply inside affected tissues. Thus, in bladder cancer, SYMPHONY demonstrated significant superiority over anti-PDL-1 monotherapy for both primary and metastatic tumors [119]. Moreover, after this therapy, tumor-bearing mice showed a long-term memory immune response capable of protecting against cancer relapse long after mice treatment. SYMPHONY therapy gave similar results also in a GBM mice model. However, the mechanism of this synergic effect is still unknown [120].

Recently, Huang et al. demonstrated that PTT treatment based on liposome platform encapsulating ICG enhanced the effectiveness, leading to the suppression of primary tumors [121]. However, this nanocarrier-mediated PTT showed only a minimal effect on the inhibition of distant tumor growth in two different colon cancer animal models (CT26 and MC38). It has been ascertained that TIM-3 and PD-1 are co-expressed in more severe T-cell exhaustion [122,123]. Studying TME, a compensatory enhancement of ICP, in tumor-infiltrating CD8 T cells has been confirmed. Combining PTT with both anti-PD-1 and anti-TIM-3 antibodies, Huang and coworkers observed the inhibition of distal tumor growth, in addition to the suppression of primary tumors, in the MC38 animal model [121].

Lastly, efficient chemo-photoimmunotherapy was achieved in HCT116 and MC38 colorectal cancer-bearing mice models through an innovative plug-and-play nanoplatform approach based on black phosphorus nanosheet (BP-NS) [124]. Beside biodegradability and biocompatibility features, the BSNS texture exhibited a high drug adsorptive capability. Moreover, it showed a strong effectiveness in converting NIR to heat; this key feature accounts for a boost in photothermal-induced DOX release and a subsequent ROS production upon NIR irradiation, thus achieving an enhancement of cytotoxic effect (apoptosis/necrosis) in HCT116, HT29 and MC38 tumor cells, mediated by ROS. In addition, this therapeutic approach also interfered with PD-1/PD-L1 pathway-regulated immune tolerance and suppression of CD8^+^ T cells, inducing the cancer cell lysis effect mediated by IFN-γ and TNF-α production. Another important achievement was maturation of DCs elicited by the photo-stimulation, which further ameliorated the lysis effect through promotion of T-cell infiltration and exemplified the immune system multi-activation potentiality of these nanoplatforms. These multiple therapeutic effects were demonstrated in both C57BL/6 and Balb/c nude mouse models, with the survival period of the treated group being significantly prolonged.

**Figure 3:**
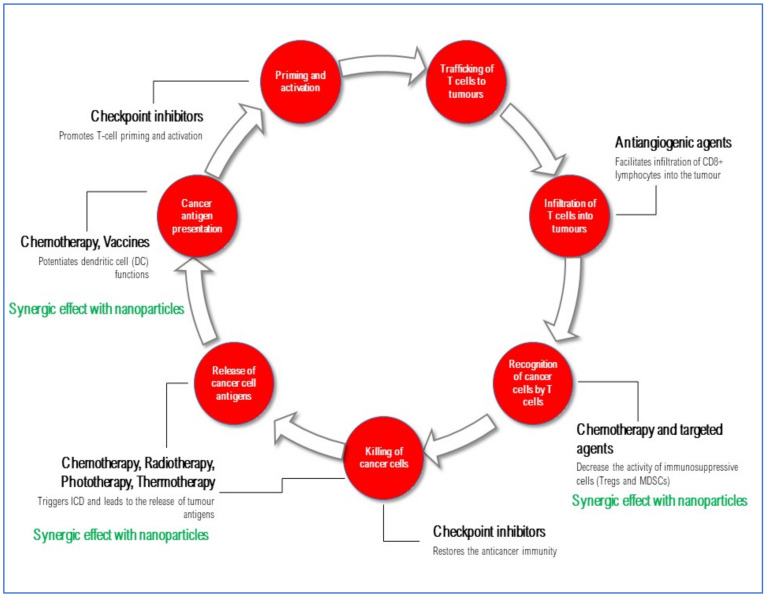
Contribution of nanoparticle-based anti-tumor strategies to the cancer-immunity cycle. Several therapeutic options currently available for the treatment of solid tumors may enhance the immunogenicity of the tumor microenvironment, thus providing a rationale for the development of potentially efficacious combination approaches. Nanoparticles may further strengthen the efficacy of these agents by increasing their effect at the tumor level.

Overall, the presented data demonstrate that nanoparticle-based strategies can strengthen the efficacy of several combination approaches for solid tumor treatment, acting at different points of the cancer-immunity cycle by enhancing the immunogenicity of the tumor microenvironment (Figure 3, Table 1).

## 5. Moving from Working Hypothesis to Clinical Trials

Some major ICI therapy limitations are uncontrolled release and poor retention at the tumor site. Nanomedicine allows for the design of a combination strategy aimed at controlling drug delivery at the target site and promoting the gradual release of drugs [125,126], thus enhancing the therapeutic effect and limiting systemic toxicity [126,127,128]. As a consequence, combination strategies of nanoparticles and ICIs are clinically sound.

Based on findings of a randomized trial, the nanoparticle albumin-bound paclitaxel (nab-paclitaxel) and the anti-PD-L1 atezolizumab have now entered the therapeutic landscape of breast cancer and are currently available for use in daily clinical practice.

The IMpassion130 study compared nab-paclitaxel plus atezolizumab or placebo in patients affected by metastatic triple negative breast cancer previously untreated for the metastatic disease and with a disease free interval >12 months. The study had two co-primary endpoints, progression-free and overall survival, and a planned hierarchical approach was planned to look at the intention-to-treat (ITT) population first, and then to the PD-L1-positive subgroup as determined by means of immunohistochemistry [129].

A significant advantage was reported in the ITT population in terms of progression-free survival with an absolute gain in median progression-free survival of 1.7 months, with no significant difference in terms of overall survival. When looking at the PD-L1-positive subgroup, a significant advantage with the addition of atezolizumab to nab-paclitaxel was evident in terms of both progression-free (with an absolute gain of 2.5 months) and overall survival (with an absolute increase of 7.5 months) [130]. The combination was well-tolerated with low incidence of grade > 2 adverse events and the expected occurrence of immune-related toxicities, thus leading to support of a positive estimation of the risk/benefit balance in favor of adding atezolizumab to upfront nab-paclitaxel, and establishing a new standard for this subgroup of patients at poor prognosis. Based on these results, atezolizumab is now approved both in Europe and in the USA in combination with nab-paclitaxel for this indication.

Of interest, other trials investigating the role of checkpoint inhibitors in the same setting failed to report consistent results. The Impassion131 study compared PTX plus atezolizumab or placebo in a population of metastatic triple negative breast cancer patients similar to those enrolled in the Impassion130 trial. Again, progression-free and overall survival were co-primary endpoints, and a reverse sequence hierarchical approach was planned, to look at the PD-L1-positive subgroup first and then at the ITT population. However, no benefit from the addition of atezolizumab to PTX was found either in the PD-L1-positive or in the ITT population [131].

A potential explanation of these disappointing results lies in the chemotherapy partner, thus leading a differential immune-modulating effect of nab-paclitaxel versus PTX to be hypothesized. The need for steroid premedication with PTX but not with nab-paclitaxel, potentially interfering with the efficacy of checkpoint inhibitors, might also be taken into regard when interpreting results from these trials.

Moreover, another study investigating the use of pembrolizumab in combination with a variety of options of chemotherapy in the upfront treatment of triple negative metastatic breast cancer patients, the Keynote-355 study, reported a significant improvement in terms of progression-free survival in the PD-L1-positive subgroup that seemed particularly relevant when the anti-PD-1 was associated with taxane-based regimens, largely consisting in nab-paclitaxel [132].

Based on the results of the phase III randomized IMpower130 study, the addition of atezolizumab to first-line carboplatin and nab-paclitaxel provided a significant survival advantage in patients with advanced or metastatic non-squamous *EGFR* and *ALK* wild-type non-small-cell lung cancer. The benefit from the addition of atezolizumab in terms of both progression-free and overall survival was independent of PD-L1 expression levels, and based on these results, this combination is currently approved both in Europe and in the USA [133].

While the combination of checkpoint inhibitors and nanomolecules has entered the clinical scenario in the treatment of breast and non-small-cell lung cancer, these strategies are currently under investigation in several therapeutic settings (Table 2).

## 6. Conclusions

Immune checkpoint inhibitors are the foremost reason for the current enthusiasm behind cancer immunotherapy. However, as increasing numbers of checkpoint-targeting molecules are entering the clinical practice, and new combinations are under investigations, several objectives may be identified for the next generation of clinical trials in this field:

- To increase the efficacy of immunotherapy in tumors already sensitive to ICIs, by preventing or overcoming mechanisms of acquired resistance;
- To optimize the tolerance to the treatment by reducing the burden of immune-related toxicities;
- To make immunotherapy, and in particular of ICIs, a treatment option also in tumors with immune-desert or immune-excluded microenvironments, developing biologically sound combinations.

Employing nanotechnology to deliver ICIs, exploiting robust knowledge acquired in decades of studies in the usage of NPs for protein delivery, can represent a solution to the above-mentioned issues. The advantages of employing nanotechnology rely on the unique nanoscale properties of carriers, workable adaptation of the carrier size, highly modulable morphological and surface properties and on the possibility to add targeting moieties.

First, based on the EPR effect, which can also be observed in some patients with advanced cancer, NPs favorably collect within tumors due to their leaky vessels and limited lymphatic drainage. In addition, though the underlying molecular mechanisms still need to be elucidated, recent evidence suggests that NPs can also enter solid tumors by active trans-endothelial processes, particularly notable for human tumors showing rather weak EPR. These various mechanisms of entry and accumulation will be relevant to overcome some of the critical points of ICI therapy, such as the localized and controlled ICI release, availability and ICI stability after infusion; further, and very relevantly, they may allow for a reduction of ICI dose and control over AIEs.

Second, nanocarriers can also be designed as intelligent platforms for controlled drug release reacting to the different stimuli present in the TME, a feature that is expected to further increase the therapeutic efficacy of nanoformulations.

Third, as checkpoint inhibitors targeting the principal inhibitory axes alone do not elicit adequate response in a vast proportion of patients carrying poorly immunogenic tumors, a combination of ICIs with nanotechnology-driven immunostimulatory treatments, such as nano-chemo/photo/thermo therapies, can help breaking immune tolerance locally and enhance systemic antitumor immunity, thus expanding the proportion of cancer patients that can benefit from these treatments.

Clearly, in this field nanomaterials go beyond the concept of an adjuvant or formulation and should be integrated into next generation ICI immunotherapies in order to improve their efficacy and reduce their toxicity. At the same time, further efforts are needed to identify the most efficacious protocols for application of these new immunotherapy agents or combination treatments, in terms of administration periods and intervals, possible off-target potentials and side effects, in order to meet the expectation of increasing the proportion of successfully treated patients.

## Figures and Tables

**Table 1:** Preclinical studies concerning NP-combined therapy plus ICI to investigate synergistic strategies in order to improve the anticancer therapeutic effects of ICI.

NPs w or w/o Adjuvant	Therapeutic Strategy	ICI	Type of Cancer	Advantages and Therapeutic Effects	Ref.
**PLGA-ICG-R837**	PTT	anti-CTLA4	4T1 breast cancer in mice model CT26 colon cancer in mice model	- efficacy of ICI in metastasis treatment - reduced mortality - increased CD8+ T cells and reduced Tregs infiltration in the secondary tumors - induction of immunological memory - reduced system toxic effects	[105]
**UCNP-R837-chlorin e6**	PDT	anti-CTLA4	CT26 colon cancer in mice model	- reinforced antitumor efficacy - increased long-term immune memory response	[106]
**PLGA-R837@Cat**	X-ray	anti-CTLA-4	CT26 colon cancer in mice model 4T1 breast cancer in orthotopic mice model	- elimination of primary tumors and disappearance of secondary tumor - increased survival - increased CD8+ T cells - decreased CD4+Tregs	[108]
**IONP**	PTT	anti-CTLA-4	4T1 breast cancer in mice model	- reduced Tregs immunosuppression	[109]
**Micelles**	Chemotherapy (epirubicin)	anti-PD-1	GBM in mice model	- increased immunogenic cell death (ICD) - decreased MSDCs and PD-L1 expression on cancer cells	[111]
**Nanodiscs**	Chemotherapy (DOX)	anti-PD-1	MC38 and CT26 colon cancer in mice model	- increased tumor regression and protection against cancer relapse	[112]
**DHA**	Chemotherapy (OXL)	anti-PD-L1	MC38 and CT26 colon cancer in mice model	- eradication and/or increased long-term tumor control	[113]
**NCP@pyrolipid**	Chemotherapy (OXL) PDT	anti-PD-L1	HT29 colon cancer in subcutaneous xenograft mice model CT26 colon cancer in mice model	- reduced tumor area - increased CD8+ T-cell infiltrations in tumors	[114]
**ZnP@pyrolipid**	Chemotherapy (OXL) PDT	anti-PD-L1	4T1 and TUBO breast cancer in mice model	- eradication of primary tumor - inhibition of untreated distant tumors and prevention of metastasis	[116]
**PC7A polymer**	Vaccines	anti-PD-L1	Melanoma in mice model MC38 colon cancer in mice model	- increased survival	[118]
**Gold nanostars**	PTT	anti-PD-L1	Bladder cancer in mice model GBM in mice model	- thermic death of tumor cells - long-term memory immune response - inhibition of cancer relapse	[119,120]
**ICG-liposome**	PTT	anti-PD-1 anti-TIM-3	MC38 and CT26 colon cancer in mice model	- inhibition of distal tumor growth - suppression of primary tumors in M38 tumor model	[121]
**BP-NSs**	Chemotherapy (DOX) PTT	anti-PD-1/PD-L1	HCT116 and MC38 colon cancer in mice model	- increased survival	[124]

**Table 2:** Clinical trials currently recruiting patients for treatment with immune checkpoint inhibitors (ICIs) combined with nanoparticle strategies.

DRUG	Phase-Indication	Nanocompound	NCT Number
HLX10	Phase II—advanced cervical cancer—second-line	Nab-paclitaxel	NCT04033354
Pembrolizumab	Phase II—HER2-negative metastatic breast cancer	Nab-paclitaxel	NCT02752685
Pembrolizumab	Phase II—advanced urothelial cancer (ABLE)	Nab-paclitaxel	NCT03240016
Atezolizumab	Phase III—PD-L1+ TN metastatic breast cancer—first-line	Nab-paclitaxel or paclitaxel	NCT04148911
Atezolizumab	Phase II—TNBC neoadjuvant	Nab-paclitaxel	NCT02530489
Atezolizumab	Phase III—Stage II, IIIA or select IIIB NSCLC (Impower030)—neoadjuvant	Nab-paclitaxel or other chemotherapy	NCT03456063
Nivolumab + Ipilimumab	Phase I/II—locally advanced pancreatic cancer	Nab-paclitaxel + gemcitabine → radiotherapy	NCT04247165
Nivolumab	Phase II—early stage NSCLC	Nab-paclitaxel + carboplatin	NCT04015778
Nivolumab	Phase I/II—borderline resectable and locally advanced pancreatic cancer	BMS-813160 + Nab-paclitaxel + gemcitabine	NCT03496662
Atezolizumab	Phase II—first-line metastatic TNBC or renal cell carcinoma	Nab-paclitaxel + IPI-549	NCT03961698
Durvalumab	Phase I—multiple solid tumors	Naoparticle-mRNA-2416	NCT03323398
Durvalumab	Phase II—head and neck cancer resected after induction chemotherapy	+/− Chemoradiation	NCT03606967
Nivolumab	Phase 1—multiple solid tumors	NBTXR3 + radiotherapy	NCT03589339

## Data Availability

Data sharing not applicable.

## Abbreviations

AIEs	Adverse immune-related events
ALK	Anaplastic lymphoma kinase
AML	Acute myeloid leukemia
ATRA	All-trans retinoic acid
BP-NS	Black phosphorus nanosheet
BBB	Blood brain barrier
CAR	Chimeric antigen receptor
Cat	Catalase
CTCAE	Common Terminology Criteria for Adverse Events
CTLA-4	Cytotoxic-T-lymphocyte-associated protein 4
DC	Dendritic cell
DHA	Dihydroartemisinin
DOX	Doxorubicin
EGFR	Epidermal growth factor receptor
EPR	Enhanced permeability and retention
FDA	Food and Drug Administration
FOXP3	Forkhead box P3
GBM	Glioblastoma
GOx	Glucose oxidase
GSH	Glutathione
ICD	Immunogenic cell death
ICG	Indocyanine green
ICI	Immune checkpoint inhibitor
ICP	Immune checkpoint protein
INFɣ	Interferon ɣ
ITT	Intention-to-treat
LAG-3	Lymphocyte-activation gene 3
mAb	Monoclonal antibodies
MDSCs	Myeloid-derived suppressor cells
MHC	Major histocompatibility complex
MMP	Matrix metalloprotease
mTOR	Mammalian target of rapamycin
NCP	Nano-scale coordination polymer
NIR	Near-infrared
NK	Natural killer
NPs	Nanoparticles
OXL	Oxaliplatin
PD-1	Program death 1
PD-L1	PD-ligand 1
PDT	Photodynamic therapy
PEG	Polyethylene glycol
PI3K	Phosphatidylinositol-3-kinase
PLGA	Poly (lactide-o-glycolic) acid
PMLA	Poly(β-L-malic acid)
PTEN	Phosphatase and tensin homologue
PTT	Photothermic
PTX	Paclitaxel
ROS	Reactive oxygen species
STAT5	Signal transducer and activator of transcription 5
TGF-β R1	Transforming growth factor-β receptor 1
TIGIT	T cell immunoreceptor with immunoglobulin and immunoreceptor tyrosine-based inhibitory motif domains
TIM-3	T cell immunoglobulin mucin-3
TME	Complex tumor microenvironment
TNF-α	Tumor necrosis factor-α
Tregs	Regulatory T cells
Zap 70	Zeta-chain-associated protein kinase 70
